# Circularly Polarized Dual-Band LoRa/GPS Antenna for a UAV-Assisted Hazardous Gas and Aerosol Sensor

**DOI:** 10.3390/mi12040377

**Published:** 2021-04-01

**Authors:** Fermín Mira, Xavier Artiga, Ignacio Llamas-Garro, Francisco Vázquez-Gallego, Jesús Salvador Velázquez-González

**Affiliations:** Centre Tecnològic de Telecomunicacions de Catalunya (CTTC/CERCA), Av. Carl Friedrich Gauss 7, Castelldefels, 08860 Barcelona, Spain; xavier.artiga@cttc.es (X.A.); ignacio.llamas@cttc.es (I.L.-G.); francisco.vazquez@cttc.es (F.V.-G.); jvelazquez@cttc.es (J.S.V.-G.)

**Keywords:** wireless sensor network, GPS, LoRa, patch antenna, circular polarization

## Abstract

UAV assisted wireless sensor networks play a key role in the detection of toxic gases and aerosols. UAVs can be used to remotely deploy sensor nodes and then collect gas concentration readings and GPS positioning from them to delimit an affected area. For such purpose, a dual-band communication system is required, supporting GPS reception, and sensor reading data transfer, which is chosen to be at 2.4 GHz using LoRa physical layer. In this work we propose a switched-beam antenna subsystem for the sensor nodes capable not only of satisfying the dual band requirements but also of maximizing communication range or energy consumption through a good antenna polarization match and improved antenna gain. This antenna subsystem is built using dual-port, dual-band, circularly polarized antenna elements, whose design and experimental validation is carefully detailed. A low profile microstrip stacked structure has been used to obtain return loss in both bands better than 15 dB, axial ratios below 1.5 dB, and wide −3 dB beamwidths around 90° and 75° for GPS and 2.4 GHz bands, respectively, thus limiting the gain reduction of the switched-beam system in critical sensor orientations. Special attention has been paid to reduce the coupling between both ports through the optimization of the relative placement of both patches and their feeding points. The measured coupling is below −30 dB.

## 1. Introduction

The use of toxic chemical warfare agents—e.g., sarin, mustard gas, or novichok—in both military conflicts and terrorist attacks [1,2] during the late 20th and early 21st centuries, revealed that such toxic chemical compounds represent a serious threat to life, not only in the context of chemical warfare but also for the civilian population. Therefore, there is an urgent need for sensing systems capable of detecting the presence and concentration of the toxic chemical compounds to delimit an exclusion area, which must be first evacuated and then monitored or decontaminated. Due to its inherent dangerousness, these sensor networks must be not only remotely operated but also remotely deployed. In addition, since the area of the attack cannot be a priori predicted, a very fast deployment is also required.

**Figure 1:**
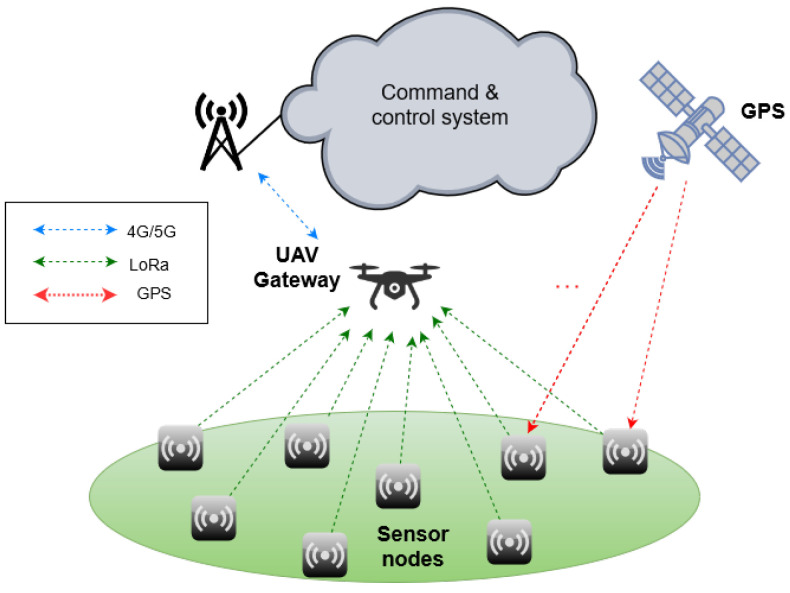
SensorQ system architecture.

In response to these requirements, we are developing the SensorQ system, a UAV (unmanned aerial vehicle)-assisted wireless sensor network (WSN) devoted to the remote detection of hazardous gases and aerosols. The operation of the SensorQ system considers that after an attack, sensor nodes are deployed on ground with the UAV and once positioned, they start reporting gas concentrations and GPS coordinates to the UAV using LoRa modulation at 2.4 GHz. In turn, the UAV acts as a gateway for the WSN, and forwards the gathered data to the command and control system. Figure 1 depicts a diagram of the SensorQ system.

Although there has been a growing interest on UAV-assisted WSNs [3], which has yielded to a vast literature on several aspects of the topic, scientific works directly detailing practical deployments and more specifically, giving details on the antennas used for the sensor nodes, which constitute a key component for the communication link, are quite scarce. Experimental works on WSNs are often based on commercial motes that use either built-in chip antennas or external dipole antennas, thus providing low gain omnidirectional patterns (e.g., [4]). However, directional antennas with higher gains [5] and polarization match [6] provide significant improvements in terms of energy consumption, enlarged communication range, and even security. This comes at the price of a higher complexity due to the need of scanning the desired sensor node field of view (FoV). Complexity reduction can be achieved by performing a discrete scanning in the form of switched-beams, which for limited FoVs can be done using conventional [7] or parasitic antenna arrays [8]. Larger FoVs require 3D structures fitting antenna elements with different spatial orientations, as proposed for the case of pure terrestrial WSNs [9,10], in which the FoV was 360° in azimuth. It must be noted that in the SensorQ system, the orientation of the sensors deployed from the UAV cannot be controlled, so the desired FoV becomes a whole sphere.

Focusing on the antenna elements forming the switched-beam system, the requirements include compactness for easy integration with the sensor electronics, moderate gain, dual-band operation (i.e., GPS L1 band at 1.5631.587 GHz and LoRa band at 2.40–2.48 GHz), and circular polarization in order to reduce polarization losses (here it is assumed that the UAV transmits in circular polarization also) and interferences due to reflected signals, in the case of GPS signal reception. A simple yet effective solution is based on the use of two microstrip patches in stacked configuration, each of them resonating at one of the desired bands. Two main structures have been proposed in the literature: (i) locating the larger patch below the smaller patch, thus acting as a ground plane for the later [11,12,13,14]; (ii) locating the larger patch on top, with a hole in the middle that permits the radiation of the smaller patch without disturbances [15,16]. In addition, such systems have been studied with single [11,12,14,15,16] or dual-port [13,14] (i.e., separated ports for each band) configurations, for linear [11] and circular polarization [12,13,14,15,16], and for the latter case, considering single [12,15], and multiple patch excitations [13,14,16].

In this paper, we introduce the antenna subsystem for the SensorQ sensor nodes and detail the design of the antenna elements in which it is based. In particular, the antenna subsystem follows the switched-beams approach of [9], but modified to provide coverage to the whole sphere using the six faces of a hexahedron. Besides, we propose a design based on dual-band circularly polarized elements instead of the single-band linearly polarized solution used in [9]. Remarkably, under this configuration, the antennas can be perfectly integrated with the sensor node electronics as part of its housing. Regarding the elements themselves, we resort to stacked microstrip patches locating the larger patch below. A dual-port solution is chosen because it offers a more direct integration with the sensor node electronics since it does not need diplexing circuits. In turn, we resort to dual-fed patches to generate the circular polarization, because they provide larger axial ratio bandwidth. Although the concept is similar to those proposed in [13,14], several differences must be highlighted. First, we provide a detailed analysis of the isolation between ports, which is deemed important here to avoid any spurious radiation from the LoRa transmitter affecting the sensitive GPS receiver. In particular, we carefully design the relative position of the patches and their feeds to reduce the coupling between them. Second, we locate the high frequency patch on the top layer so that a moderate low dielectric constant substrate can be used for both patches, what results in similar bandwidth and gain results but at reduced antenna profile. Finally, we use microstrip technology for the feeding network, facilitating its fabrication and the soldering of the Wilkinson divider resistors.

The rest of the paper is organized as follows. Section 2 introduces the sensor node and the antenna subsystem, Section 3 details the design of the dual-band circularly polarized elements, Section 4 presents simulation and measurement results, and finally Section 5 concludes the study.

## 2. Sensor Node

The SensorQ system is sketched in Figure 1. It consists of a set of sensor nodes deployed by the UAV; the UAV itself acts as a gateway of the WSN and thus bridges the sensor nodes with the command and control system, which in turn collects and processes the sensor node readings. Next, we introduce the sensor node as previous step for discussing the antenna subsystem of the sensor node, which is detailed in Section 2.1.

The sensor node is composed of a micro-controller that controls all its functions; the LoRa radio transceiver at 2.4 GHz; four sensors including the hazardous gas and aerosol sensor, a GPS receiver, a three-axis accelerometer and an altimeter to measure the altitude and temperature; the dual-band LoRa/GPS antenna subsystem, and the power module consisting of batteries and a voltage regulator. The prototype of the sensor node is built using commercial of the shelf components except for the antenna subsystem, which is the main object of discussion in this paper, and a novel low-cost sensor head for the detection of hazardous chemical compounds in aerosol or gas state. The sensor head is based on the surface plasmon resonance effect using a micromachined Otto chip [17], integrated with a compact reflectometer [18]. The Otto chip can offer some advantages over the traditional Kretschmann configuration as discussed in [19]. The LoRa radio transceiver and microcontroller are the SX1280 by Semtech (Camarillo, CA, USA) and the STM32L152RE by STMicroelectronics (Geneva, Switzerland).

Though still under development, the form factor of this version of the sensor node is assumed to be an irregular hexahedron with faces of area in the order of 10 × 10 mm.

### 2.1. Sensor Node Antenna Subsystem

The concept behind the antenna subsystem of the sensor node is to exploit its current dimensions in order to deploy a directional switched-beam system, which would allow improving the communication performance with respect to miniaturized chip antennas. In particular, it could reduce power consumption, thus extending the sensor nodes’ battery lifetime; or extending the communication range, enabling data collection at longer distances, which provides advantages in terms of coverage area. Besides, it would also secure good position readings from the GPS receiver. As matter of example, the LoRa radio chip SX1280 announces a current consumption of 24 mA and 10 mA for output powers of 12.5 dBm and 0 dBm, respectively. Therefore, an antenna gain improvement of 6 dB would result in a 33% power consumption reduction, (assuming 16 mA for an output power of 6.5 dBm). For a fixed transmit power, a 6 dB gain improvement means doubling the communication range. Taking into account the high sensitivity of the SX1280 that goes down to −132 dBm, it could allow data collection through even higher aerial vehicles such as high-altitude platforms or LEO nanosatellites, at altitudes between tenths to hundredths of kilometers. This discussion also applies to having a good polarization match between transmitters and receivers. This is the reason why we consider the use of circular polarization for both GPS and LoRa bands, which can provide up to 3 dB improvements with respect to simpler liner polarization solutions. Note here that the UAV is assumed to transmit in circular polarization.

**Figure 2:**
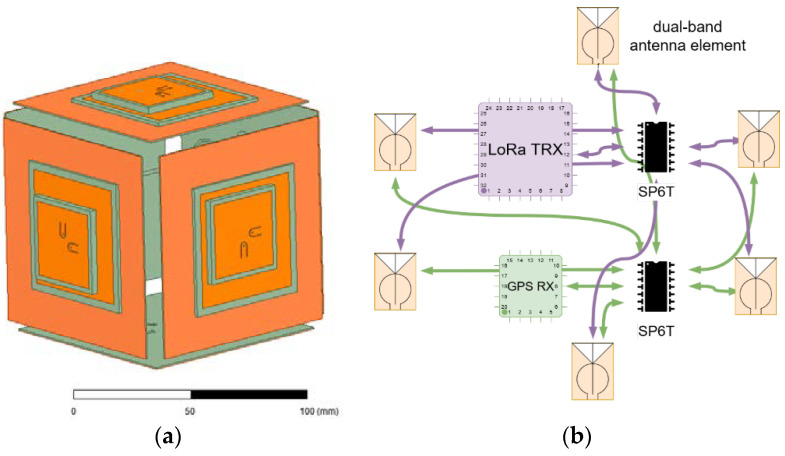
Switched-beams antenna concept: (**a**) antenna subsystem; (**b**) schematic of the switching mechanism. The hexahedron is not closed to allow the hazardous gas flowing into the sensor node.

Therefore, the proposed antenna subsystem, as shown in Figure 2 and similarly to the design in [9], consists of placing one antenna element in each face of the sensor node hexahedron and select the operating face by means of a low loss SP6T RF switch. The selection will be based on the current sensor node orientation extracted from the three-axis accelerometer and checked through the communication protocol. In this way, it is possible to secure the communication link regardless of the sensor node orientation. Note however, that the reconfiguration capabilities go beyond that, and instead of activating the face pointing to the sky, it would be possible to operate with a lateral face to permit node to node communications in case it is needed. Also note that, in this configuration, the antenna elements act also as part of the protecting housing and package of the sensor node resulting in a perfect antenna-sensor node integration.

The antenna elements are based on dual-port dual-band circularly polarized microstrip patches. The specific bands of interest are GPS L1 (1.563–1.587 GHz) and LoRa (2.40–2.48 GHz). The dual-port configuration is chosen for a direct integration with the sensor node electronics, without the need of additional diplexing circuits. Short RF cables with MCX connectors will be used to feed the patches from the output of the RF switch. Next section details the antenna element design.

## 3. Circularly Polarized Dual-Band Antenna Element Design

The proposed antenna element consists of two circularly-polarized microstrip patches stacked one on top of the other. In both cases, the circular polarization is obtained by exciting two orthogonal modes through two feeds with a phase shift of 90°. This configuration enhances the axial ratio bandwidth and its robustness against manufacturing tolerances. Separate ports for each patch are considered.

**Figure 3:**
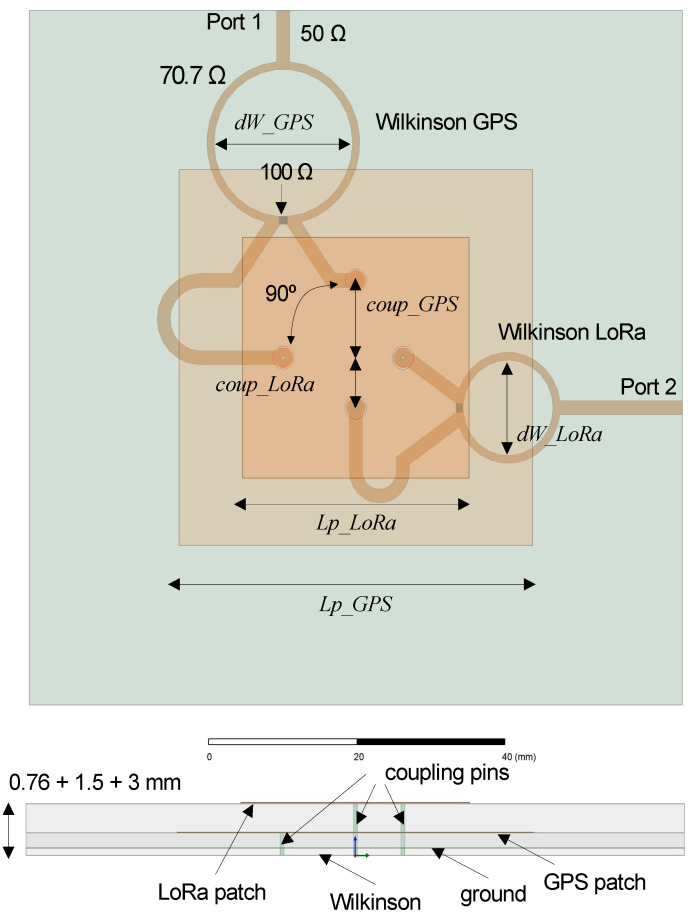
Structure of the proposed antenna element.

The antenna structure is based on a four-layer structure with an overall thickness of 5.3 mm, as depicted in Figure 3. The higher frequency patch (LoRa) is etched on the top layer over a 3 mm Taconic RF35 substrate (ε_r_ = 3.40), which allows achieving the required bandwidth. Indeed, the 3 mm substrate results from the attachment of two 1.5 mm substrates. The lower frequency patch (GPS L1 band) is etched in the second layer over a 1.5 mm Taconic RF35 substrate. Both patches are square, and their side lengths are *Lp_LoRa* and *Lp_GPS*, respectively. In this configuration, the lower patch acts as the ground plane for the higher patch; and the radiation properties of the lower patch are not significantly affected by presence of the higher patch due to its reduced dimensions. The third layer consists of a 90 × 90 mm metallic plane that acts as the ground for both the lower patch and the patches feeding network. It was chosen to provide the required directivity, but it could be reduced in case that the final size of the sensor requires smaller footprint. The feeding network is etched on the fourth layer, on a 0.76 mm Arlon 25 N substrate (ε_r_ = 3.38). It consists of two independent Wilkinson power dividers. The λ/4 sections of the two dividers are implemented as circles of diameter *dW_GPS* and *dW_LoRa*, respectively. The 90° phase shift between the two feeds of each patch is achieved by adding extra line length to one of the branches at the dividers output. The proposed configuration in Figure 3 produces right-handed circular polarization (RHCP) for the GPS band, and left-handed circular polarization (LHCP) for the LoRa band. It is easy to modify the structure to change the sense of the polarization or the placement of the ports in case there is an interest on mounting both ports on the same side or opposite sides. The two feed pairs are implemented with vertical pins going through small circular holes on the ground layer in the lower patch case and on the ground and GPS patch layers in the case of the upper patch.

The dielectric around the patches has been removed, preserving only a margin of few millimeters in order to reduce the cost and weight of the antenna, leaving bigger the thinner substrate with the power dividers and ground plane. Substrates with low dielectric constant or even air spacings achieve better bandwidth for the same thickness, but at the cost of a higher footprint. For our application, dielectric substrates with dielectric constant around 3.5 are a good choice, with a good ratio between footprint and thickness. Still, the employed substrates offer a better performance in term of losses than the commonly employed FR4. In addition, air spacings are discarded to provide mechanical robustness, since the antenna forms part of the sensor node housing.

**Figure 4:**
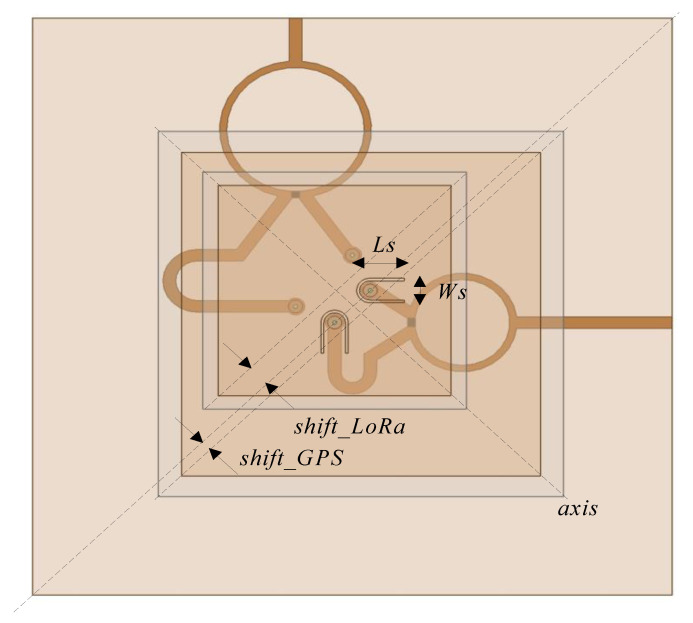
Modification of the placement of patches.

The relative position of the upper patch and its feeds with respect to the lower patch and its feeds has a significant effect on the coupling between them, which also affects the radiation properties of both patches. In order to reduce this effect, we propose a displacement of the patches on a diagonal axis, as depicted in Figure 4, combined with an optimized location for the four feeds. A parametric analysis has been performed, varying the displacement of both patches (i.e *shift_GPS* for the lower patch and *shift_LoRa* for the upper patch), and the location of the feeds in the two orthogonal axes (i.e., *coup_GPS* for the lower patch and *coup_LoRa*, for the upper patch). From this analysis we have extracted three antenna designs whose dimensions are specified in Table 1. The Antenna 1 corresponds to a centered placement of both patches, Antenna 2 considers only the diagonal displacement of the upper patch, and Antenna 3 considers the displacement of both patches. Note that in Antennas 2, and 3, the impedance matching of the upper patch feeds was improved by the insertion of two slots of size *Ls* = 6.7 mm and *Ws* = 3.8 mm with thickness 0.4 mm, as can be observed in Figure 4. The simulation for the three designs as well as the measurement of a selected one is discussed in next section.

## 4. Simulation and Measurement Results

**Figure 5:**
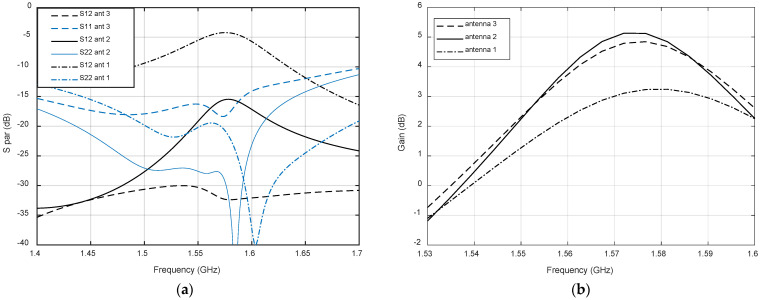
Simulated results for the three proposed antennas in the GPS band: (**a**) scattering parameters; (**b**) gain.

The three antenna designs specified in Table 1 have been simulated using Ansys HFSS. Figure 5a shows the s-parameters referred to the GPS port, i.e., the GPS return loss together with the coupling of the two ports in the lower band. Whereas return loss is quite good for the three designs, the Antenna 1, with both patches centered, shows a very strong coupling, higher that −5 dB, around the GPS band. A reduction of the coupling up to −15 dB is achieved in Antenna 2 by displacing the upper patch such that *shift_LoRa* equals *coup_LoRa.* In addition, the displacement of 1.2 mm of the lower patch in Antenna 3 provides further reduction of the coupling up to values of −30 dB, with a slightly deterioration of the input impedance matching. Figure 5b shows the simulated realized gain of the three antennas. It can be observed that for Antenna 1, the strong coupling is acompassed with an important reduction of the antenna gain. Antenna 2 shows the best gain, but with a slight difference with Antenna 3.

**Figure 6:**
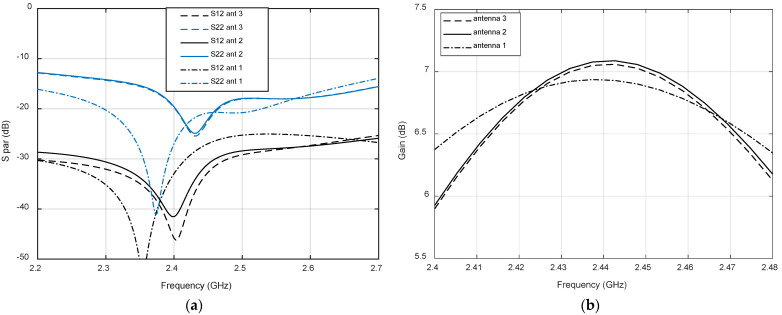
Simulated results for the three proposed antennas in the LoRa band: (**a**) scattering parameters; (**b**) gain.

Regarding the LoRa port results, depicted in Figure 6, the coupling level for all the designed antennas is good, below −25 dB in all cases. The return loss is slightly better in Antenna 1 due to the higher value of *coup_LoRa,* but in all cases is above 18 dB. The gain of Antenna 1 is slightly lower in the center of the band, but the bandwidth is slightly larger. As expected, the gain of all designs in the GPS band are lower than the LoRa band due to the lower thickness of the patch, which has been chosen to keep a low antenna profile.

**Figure 7:**
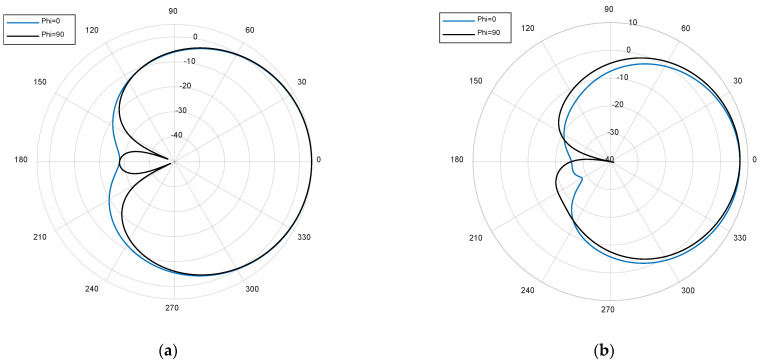
Simulated realized gain (dB) for two perpedicular planes at the central frequency (**a**) RHCP at 1.574 GHz; (**b**) LHCP at 2.44 GHz. The results correspond to Antenna 3.

Figure 7 depicts the simulated RHCP (GPS band) and LHCP (LoRa band) realized gain radiation patterns for the two perpendicular cutting planes, Phi = 0° and Phi = 90°, along the axis perpendicular to the antenna. Both radiation patterns are almost symmetrical, with only small variations for different planes, especially in GPS band. The −3 dB beamwidth for GPS band is around 88° and 77° for LoRa band. These wide beamwidths and uniform patterns are good for our application, limiting the gain loss in the critical case in which the UAV is just located between two faces of the sensor node hexahedron.

**Figure 8:**
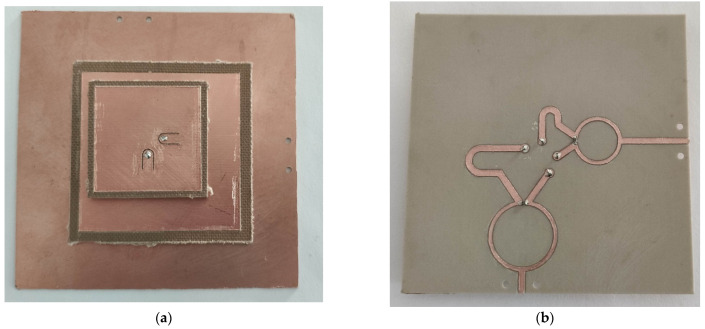
Fabricated antenna (**a**) top view; (**b**), bottom view.

After the evaluation of the simulated results, we have decided to fabricate and measure Antenna 3, which shows the best performance in terms of mutual coupling, gain, and input impedance match. Figure 8 shows the implemented antenna, fabricated with an LPKF milling machine. All the substrates were mounted by hand in our laboratory.

**Figure 9:**
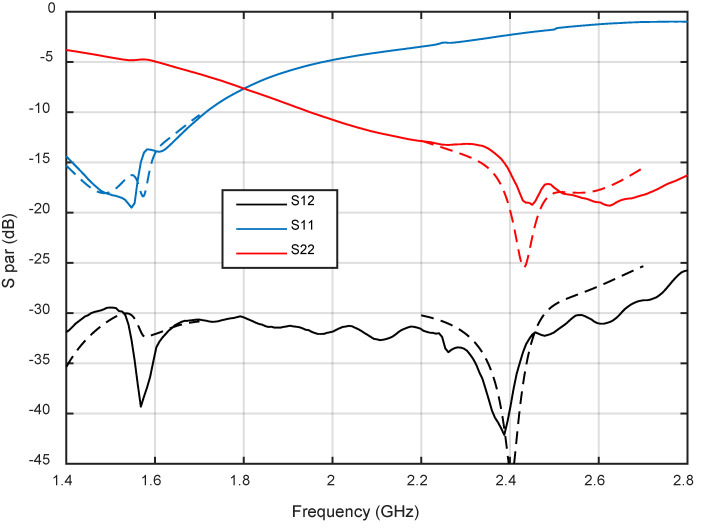
Simulated and measured scattering parameters. Solid line for mesured results and dashed line for simulated results.

**Figure 10:**
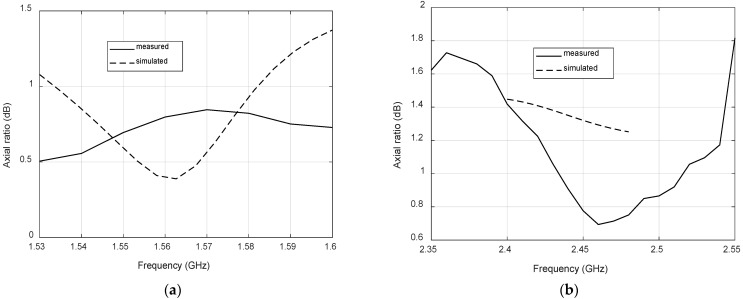
Simulated and measured axial ratio: (**a**) GPS band; (**b**) LoRa band.

Figure 9 shows the measured s-parameters. A very close agreement between simulations and measurements is observed in the bands of interest, resulting in coupling values below −30 dB and return loss above 15 dB. Figure 10 compares simulated and measured axial ratio at the antenna boresight for both bands. Again, a reasonable agreement is observed, with measured axial ratios below 1 dB and 1.5 dB for GPS and LoRa bands, respectively. These low values and the wideband response are the result of feeding the patches with a dual-fed configuration based on Wilkinson power dividers.

**Figure 11:**
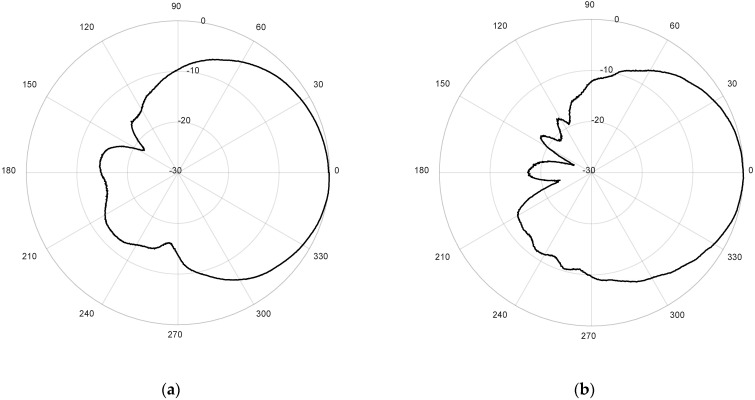
Measured radiation pattern at plane Phi = 0° normalized to zero (dB): (**a**) 1.574 GHz; (**b**) 2.44 GHz.

Finally, the measured radiation patterns of the antenna for both ports are presented in Figure 11, for the cutting plane Phi = 0° and normalized to zero. The antenna has been measured in the anechoic chamber by using the same reference antenna employed for the axial ratio measurements. The measured −3 dB beamwidths are 75° for the LoRa band, and 93° for the GPS band, showing again a good agreement with simulation results.

## 5. Conclusions

A low profile dual-port dual-band circularly polarized antenna element based on microstrip stacked patches has been designed and measured. Very good agreement between simulation and measurement results have been obtained. A dual-fed patch configuration based on Wilkinson dividers provided a wideband response both for input impedance matching and axial ratio. The placement of the patches and feeding pins is optimized to reduce the coupling between the two bands below −30 dB. These antenna elements are the basis of a switched-beam antenna subsystem for a hazardous gas and aerosol sensor node, which has been also introduced. This solution permits improving the communication range and energy consumption of the sensor node with respect to a solution based on miniaturized omnidirectional chip antennas. In addition, it provides a perfect integration of the antenna subsystem with the sensor node since it becomes part of its housing and package.

## Figures and Tables

**Table 1:** Main dimensions (mm) of the designed antennas.

Variable	Antenna 1	Antenna 2	Antenna 3
*dW_GPS*	9.55	9.68	9.68
*dW_LoRa*	6.5	6.7	6.7
*coup_GPS*	10	8	8
*coup_LoRa*	7	5	5
*Lp_GPS*	48.7	50.5	50.5
*Lp_LoRa*	31.2	32.8	32.8
*shift_GPS*	0	0	1.2
*shift_LoRa*	0	5	5

## Data Availability

Data available at the CTTC.
